# Commentary: the value of PrEP for people who inject drugs

**DOI:** 10.7448/IAS.19.7.21112

**Published:** 2016-10-18

**Authors:** Rosalind L Coleman, Susie McLean

**Affiliations:** 1 UNAIDS Office of the Science Panel, Geneva, Switzerland; 2 International HIV/AIDS Alliance, Brighton, UK

**Keywords:** PrEP, people who inject drugs, HIV prevention

## Abstract

**Introduction:**

The offer of pre-exposure prophylaxis (PrEP) is recommended as an additional option for HIV prevention for people at substantial risk of HIV infection as part of combination HIV prevention approaches. Implementing this depends on integrating PrEP in public health programmes that address risky practices with evidence-based interventions, and that operate in an enabling legal and policy environment for the delivery of health services to those at higher risk of HIV infection. What does this recommendation mean in terms of the diverse range of HIV prevention needs of key populations, some of whom are so discriminated against that they exist essentially outside formal systems such as national public health services, and for whom a substantial risk of HIV is part of a larger adverse and hostile situation? We discuss this question with reference to people who inject drugs, informed by concerns and comments that emerged from a series of consultations.

**Discussion:**

HIV prevention is part of a spectrum of injecting drug users’ priorities, and their access and uptake of HIV prevention services is contingent on their wider “risk environment.” The need to address structural barriers to services and human rights violations, and to improve access to comprehensive harm reduction programmes are of prime importance and would have higher value than a mono-focus on HIV prevention. Where existing harm reduction activities are inadequate, fragile or dependent on external donors, shifts in funding priorities, including, for example, towards PrEP, could threaten investment in the broader programmes. For these reasons, it cannot be assumed that PrEP promotion will always be supported by people who inject drugs.

The sexual partners of people who inject drugs, non-opioid users who also inject and for whom there is no established substitution treatment, as well as drug users who are unable to negotiate safe sex may value PrEP. As for all key populations, the involvement of people who inject drugs in shaping services for their consumption is vital and too often ignored.

**Conclusions:**

For people who inject drugs and who experience discrimination, violence or harassment, implementation of PrEP should be guided by understanding and engaging with their interconnected range of needs, risk practices, priorities and options. The differentiated needs of sub-populations that inject a range of drugs, and their sexual partners, require further exploration.

## Introduction

HIV prevention is currently insufficient for many populations who inject drugs and who continue to bear a disproportionate burden of HIV [1]. Across all regions, the prevalence of HIV for people who inject drugs is up to 50 times the rate of the rest of the adult population and people who inject drugs account for 30% of all new HIV infections outside sub-Saharan Africa [1,2]. This inadequacy of HIV prevention is most prominently illustrated in Eastern Europe and Central Asia, where new HIV infections are up 57% compared with 2010 [1] and more than half of these new infections are among people who inject drugs.

Pre-exposure prophylaxis (PrEP) can effectively prevent HIV when introduced in an enabling combination HIV prevention programme and chosen by people at high risk of infection [3,4]. The integration of PrEP into public health programmes has been presented as an opportunity to strengthen HIV prevention for key populations when implemented in a context of linked action for human rights, supportive laws and violence prevention [5,6]. PrEP is recommended as a choice for people who inject drugs by the American Centres for Disease control [7] and in the recent WHO recommendation [5], but some critiques of the value and or effectiveness of PrEP for people who inject drugs are emerging, including from consultations with people who inject drugs [8,9]. These consultations, co-ordinated by the International Network of People who Use Drugs (INPUD), brought together 75 representatives of people who inject drugs from 33 countries, predominantly from Eastern Europe and Central and Eastern Asia to discuss policy and programme considerations of PrEP in their situations. The implementation challenge is to design PrEP strategies that are context specific and differentiated for key populations in all their diversity, including for people who inject drugs [10].

The risk of HIV transmission for people who inject drugs exists in a context of other adverse health events and the high HIV rates reflect their broader health problems that include hepatitis C (HCV) [11] and other blood-borne diseases, overdose, vein damage and tuberculosis [12]. Much of the health risk associated with injecting drug use is exacerbated by poverty, inequality, criminalization, violence and discrimination [13].

WHO, UNAIDS and UNODC have defined and endorsed a range of harm reduction interventions to address and prioritize HIV and related health needs of people who inject drugs [14]. Key interventions include needle and syringe programmes (NSP) and opioid substitution therapy (OST) to reduce unsafe injecting and manage drug dependency, as well as the promotion of condom use, facilitated access to testing and treatment for HIV and other sexually transmitted infections, TB, and HCV, and naloxone provision to prevent overdose, supported by appropriate information, education and communication interventions, and “critical enablers,” such as supportive laws, anti-discrimination interventions, interventions to make health services more accessible and acceptable to people who use drugs and anti-violence interventions [14–16]. This set of interventions represents the formal list of evidence-based “harm reduction” interventions around which significant consensus lies as evidenced by their endorsement by international, regional and national organizations and governments [17,18, para.43]. This harm reduction approach responds to a wide range of adverse health needs, is relatively inexpensive to implement [19] and can have a high impact on HIV and HCV transmission [14,16], Despite this, only half of all countries that report injecting drug use provide OST and even fewer offer NSP [20]. Where these interventions are available, they are often provided at an insufficient scale to have a public health impact.

Lack of scale of these evidence-based interventions results from a number of factors: lack of funding and prioritization, the design and delivery of services that are not targeted to reach key populations and structural barriers that prevent people who use drugs from accessing services [1,21]. These factors shape the wider risk environments of people who inject drugs [22] and constitute a form of structural violence [23], when associated with repeated incarceration; compulsory registration on official lists as drug users; and high rates of violence, police harassment, homelessness and poverty.

People who inject drugs will need to know about, believe in and value PrEP, alongside other interventions such as clean needles and OST. Without consideration of the sometimes violent and hostile environment in which these harm reduction services are delivered, people who inject drugs may not embrace PrEP and instead view it as a reductionist and potentially destabilizing intervention that could divert attention away from existing harm reduction services.

## Discussion

Concerns expressed about PrEP by people who use drugs underline the importance of identifying particular policy environments, geographies and sub-populations of people who inject drugs and their sexual partners for whom PrEP may be of value.

### PrEP and harm reduction services

The people who inject drugs who were involved in the consultations agreed that, in a context where other key harm reduction services were in place, PrEP would be a desirable option for some people who inject drugs [9]. It was felt, however, that in countries with the highest injection-drug-associated HIV burden, the reality was far from being realized. Respondents expressed opposition to any introduction of PrEP that was not part of an effort to strengthen broader harm reduction and social justice programmes.

I don't see why it can't be part of a truly comprehensive, universally accessible package of services. But that is not the reality. So, given that, it is simply not a priority. [8, p. 10, participant from Eastern Europe and Central Asia region]

Even if we had PrEP, we would still need clean works. [8, p. 3, participant from Eastern Europe and Central Asia region]

Sustainability and expansion of harm reduction services is a much bigger priority. [8, p. 10, participant from Asia Pacific]

Any new intervention, including PrEP, should strengthen broader health promotion and combination HIV prevention approaches, bolster any existent effective interventions and respond to the diverse needs of the population [15]. A comprehensive combination HIV prevention programme includes condom and lubricant provision, immediate initiation of antiretroviral therapy and the offer of PrEP and also supports related interventions such as harm reduction programmes for people who inject drugs and voluntary medical male circumcision (VMMC) for men in eastern and southern Africa [24].

HIV prevention spending for key populations such as people who inject drugs in low- and middle-income countries is heavily dependent on international financing which is flattening while domestic funding for key populations is low [17,25,26]. A change in donor priorities could destabilize existing harm reduction programmes if they compete for limited funding [27]. This threat is intensified by weak policy and financial support by many governments who have permitted harm reduction programmes that are funded by external sources but who show no or limited commitment to funding harm reduction from national budgets when donors retreat [25,28]. In contexts where harm reduction programmes are politically unpopular, PrEP could present a convenient and “magic bullet” intervention that allows governments to claim that they are addressing HIV prevention, whilst reducing funding and policy support for other evidence-based harm reduction interventions that people who inject drugs need and prioritize [9]. The ethics of providing PrEP when HIV treatment access for people who use drugs is so poor [2,14] was also questioned at the consultations.

PrEP could give them an excuse to close down harm reduction programmes. [8, p. 8, participant from Eastern Europe and Central Asia region]

[PrEP] is entirely inappropriate, given the exceptionally low levels of access [to treatment] for people who inject drugs who live with HIV. [8, p. 4, participant from Europe]

In any situation, PrEP can only be economically and clinically effective when it is funded as part of an accessible and comprehensive HIV prevention and treatment programme [29].

### PrEP, drug use and sexual transmission of HIV

People who inject drugs account for a disproportionate share of HIV burden whether attributable to injection drug use or in combination with sexual transmission [30,31]. There is a complex and fluid interaction between the injection or other use of drugs and increased sexual HIV transmission risk [30]. People who inject drugs report low levels of condom use [31–33]. In Eastern Europe and Central Asia, where half of new infections are among people who inject drugs, 6% of new infections are among sex workers and one-third of new infections are among clients of sex workers and other sexual partners of key populations [1]. Therefore, PrEP has possible value in the social and sexual networks of people who inject drugs where the incidence of HIV is high. These people might include the sexual partners of people who inject drugs, non-opioid drug users who also inject but for whom there is no established substitution treatment and others within the wider drug-using community who are unable to negotiate safe sex, including both women and men who inject drugs and who have sex with men, people who inject drugs and sell sex, transgender people and those who are vulnerable to sexual violence or exploitation. Given what we know of the overlap [30] and double HIV burden of sex work and injection drug use [34], there is a need for synergy and a broad approach to harm reduction programming. This could include the offer of PrEP to address both sexual and injection-related health risks. The one trial of PrEP that was specific to people who inject drugs was not able to differentiate between the prevention of sexual- and injecting-related HIV transmission [35].

Consultations with these linked populations could identify additional situations where PrEP might be valuable. Some parallels may be drawn from the views of sex workers who also experience stigma and discrimination that prevents them from engaging with health services [30]. A comprehensive literature search found an “enthusiasm” to use PrEP among sex workers in China, Peru, sub-Saharan Africa and the United States if it were to be distributed through user-friendly services and promoted with non-stigmatizing information and adherence support [36]. This view is tempered with the caution that PrEP introduction should benefit sex workers and their immediate community and not be introduced only for the public health impact in the general population [37]. For some, debate on PrEP promotion provides an opportunity to engage with policy makers on human rights [38].

PrEP gives us an opportunity to advocate for sex workers’ rights …. The conversation about biomedical HIV interventions is not going away, and regardless of individual opinion, the folks at the top are still lumping us all into one category …. If they're making us a priority “to address” us as a “key population,” we should be at that table. [38, sex worker activist, Chicago]

This reaction found an echo from a North American INPUD consultation participant and emphasizes the notion that PrEP services should be in step with the varying needs and desires of the priority population for whom it is intended.

Now you might say we have other priorities, … people should have their options … And like anything else, if you're not at the table, but you're just being a [PrEP] naysayer, you're going to be left out of how these things actually get rolled out. [8, p. 10]

It is important to remember that people who inject drugs have the same sexual rights and sexual health needs as those in the general population [39] and that in contexts where HIV transmission is at least partly driven by unsafe injecting in combination with risky sex, HIV prevention impact achieved for people who inject drugs can influence HIV transmission dynamics in the wider population [40].

### Provision and uptake of PrEP

For all criminalized and highly marginalized (key) populations, an enabling environment where their voices can be heard, supported by legislation and zero tolerance for violence, is vital to meet HIV prevention targets and ameliorate many other adverse health events [2,41]. Where these conditions are still lacking, their absence is a huge constraint to achieving better health and quality of life [22,42]. Without them, improved services can remain under-utilized and inefficient since key populations will often remain underground for fear of arrest or violence. A person's request for PrEP can require the disclosure of sometimes illegal and risky practices, along with repeated contact with sometimes hostile health services. In conditions such as these, people who are at substantial risk of HIV exposure and who could benefit from PrEP are unlikely to seek it for fear of discrimination, breaches of privacy, criminal sanctions or other threats.

If we're talking about people who are targeted by evidence of prostitution or injection drug use, they are already going to have trouble accessing the level of institutional care they need to be on PrEP, so PrEP is not a solution for the people who need it most. [43], Daniel Wolfe Director, International Harm Reduction Development]

You wouldn't be able to get it without giving your name, which would be a barrier for sure. As a mother there is fear of getting children removed, fear of losing one's job. [43], p. 6, participant from Europe]

The history of rights-based health interventions, including for people who inject drugs, has advanced through a series of small victories [40]. The requirement to attend to the context, practices and priorities of key populations applies to all HIV programme implementation, and the emergence of interest in PrEP presents an opportunity to examine these factors more deeply. Current focus and debate on PrEP implementation provides an opportunity for people who inject drugs who are interested to shape the development and design of PrEP services. Health planners and decision-makers will need to engage with people who inject drugs and their sexual partners in order to understand their interest in PrEP, and how any such interest can be integrated with broader prevention services and, for example, the need for drug dependency treatment, overdose treatment, HCV and HIV treatment and the need for protection from human rights violations [2].

## Conclusions

PrEP is best understood as an additional potential HIV prevention option for some people who inject drugs and their sexual partners in specific circumstances. The challenge is to ensure that the potential of PrEP is not undermined by narrow and overly biomedical understandings of its value, removed from the real lives of those most at risk of HIV exposure. Harm reduction programmes are intended to address a range of interests and needs, and it is likely that harm reduction programmes could be an effective implementation platform for PrEP for people who inject drugs. (From data and experience of using the medicines in HIV treatment, no interaction is expected between PrEP containing tenofovir/emtricitabine or tenofovir/lamivudine and heroin, methadone or methamphetamine [44]).

PrEP can be an opportunity to harness a specific intervention for longer term public health and human rights outcomes. PrEP will realize its HIV prevention potential if it is introduced in a way that complements and strengthens existing harm reduction and health promotion activities, if it contributes to reducing discrimination and empowers key populations at risk of HIV infection. Creating opportunities to hear from and engage with people who use drugs about their interests, needs, values and preferences regarding HIV prevention, alongside wider health and other needs, is a priority for health programmers and decision-makers. These are some of the factors that will ensure the potential of PrEP for people who inject drugs is realized in a variety of real-world settings.
